# Successful Management of an “Unresectable” Intrahepatic Cholangiocarcinoma with Neoadjuvant Systemic Therapy, Chemoembolization, and Extended Hepatectomy with Portal Vein Reconstruction

**DOI:** 10.7759/cureus.2696

**Published:** 2018-05-28

**Authors:** Colin K Cantrell, Jared White

**Affiliations:** 1 UAB School of Medicine, University of Alabama at Birmingham, Birmingham, USA; 2 Surgery-Liver Transplant and Hepatobiliary Surgery, University of Alabama at Birmingham, Birmingham, USA

**Keywords:** cholangiocarcinoma, transarterial chemoembolism, extended hepatectomy, portal vein reconstruction

## Abstract

Cholangiocarcinoma is a rare, but invariably fatal primary hepatic tumor that is often diagnosed at advanced stages with minimal options of surgical cure. Relatively few patients diagnosed with cholangiocarcinoma are considered surgical candidates, owing to the extent of the disease at presentation, the presence of vascular invasion or extrahepatic disease, and/or poor functional status with advanced age being most commonly associated. There is no clear consensus for the management of advanced cholangiocarcinomas, as the current literature is typically based on limited patient numbers and anecdotal experiences at best. This case report represents the successful management of a large, advanced intrahepatic cholangiocarcinoma using multiple treatment modalities including systemic chemotherapy, liver-directed therapy, portal vein embolization, and extended hepatectomy with portal vein resection and reconstruction.

## Introduction

Cholangiocarcinoma (CCA) is a malignant tumor that arises from the epithelial lining of the biliary tree. Based on the anatomic location, CCAs are classified into three subgroups including intrahepatic CCA (iCCA), peri-hilar CCA (pCCA), or distal CCA (dCCA) [1]. All types of CCAs have been increasing in incidence for the last 30 years, with a steadily increasing mortality rate of iCCA, contrasted with a stable or slightly decreasing mortality rate of pCCA and dCCA [2]. The relative paucity of risk factors associated with CCAs makes this group of malignancies difficult to diagnose early and considerably more difficult to treat when diagnosed at the late stages of the disease process. Intrahepatic cholangiocarcinoma is the second most common primary hepatic tumor, reportedly accounting for 10-15% of primary liver cancers [3]. This case report represents the successful treatment of an advanced intrahepatic cholangiocarcinoma utilizing preoperative systemic therapy, liver-directed therapy, extended right hepatectomy, and portal vein reconstruction.

## Case presentation

**Figure 1:**
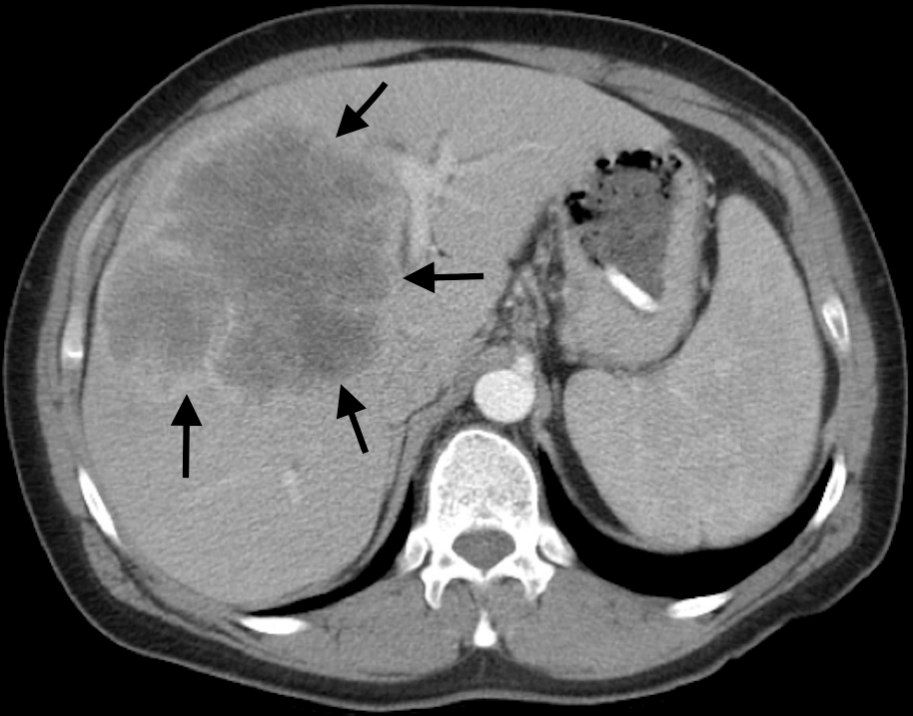
Large central cholangiocarcinoma with impingement on left portal vein bifurcation.

The patient is a 39-year-old female that presented to an outside facility with right upper quadrant (RUQ) abdominal pain. A contrast-enhanced computed tomography (CT) scan in late arterial phase showed a large 12 cm x 10 cm peripherally enhancing mass extending from the right hepatic dome to the gallbladder fossa (Figure 1). Percutaneous biopsy was done, consistent with moderately differentiated intrahepatic cholangiocarcinoma, which was CK19+, MOC31+, CA19-9+, CK7+, and CK20 negative. She had retroperitoneal adenopathy, but no extrahepatic disease in the chest, abdomen, or pelvis. She was transferred to our University of Alabama at Birmingham hospital for surgical consultation. The patient was completely functional, although she suffered from abdominal pain. On physical exam, she displayed moderate RUQ tenderness and right epigastric pain. Her laboratory workup was normal, including a total bilirubin of 0.4 mg/dL (normal range 0.3-1.2 mg/dL), normal transaminases, normal white blood cell count, normal hematocrit, and normal CA 19-9 level of 15 units/ml (normal range 0-35 units/ml). International normalized ratio (INR) was within normal limits.

During review at our institution's multi-disciplinary liver tumor board, she was felt to be "borderline" resectable for an extended right hepatectomy due to concern for inability to obtain negative surgical margins along the left portal vein and relatively small hepatic remnant by volumetric analysis due to the size and location of her tumor. Medical oncology was then consulted to consider neoadjuvant therapy in hopes of “downstaging” this lesion to a resectable situation. After this consultation, her case was again presented in our liver tumor board, and recommendations were for chemotherapy to include gemcitabine and cisplatin, as well as liver-directed therapy to include trans-arterial chemoembolization (TACE) with Irinotecan (DEBIRI). Depending on her response to therapy, the plan was for right portal vein embolization in preparation for extended right hepatectomy.

**Figure 2:**
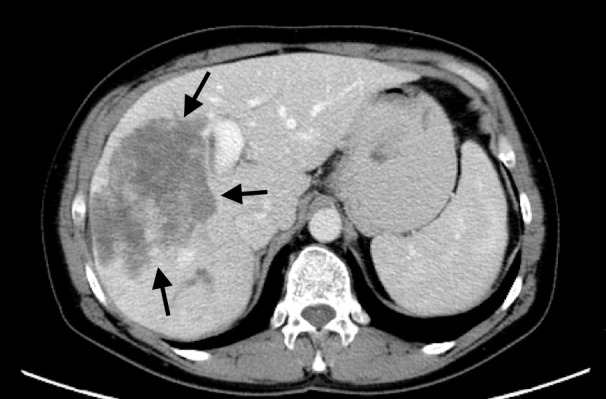
Axial image of large central cholangiocarcinoma following chemotherapy and chemoembolization.

**Figure 3:**
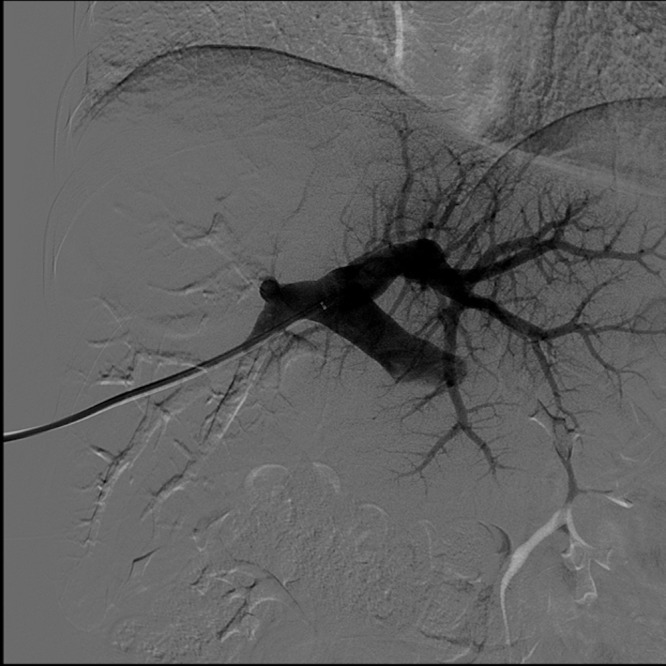
Fluoroscopic image of successful right portal vein embolization. Left lateral segment shown well perfused with contrast.

The patient received four cycles of gemcitabine/cisplatin on days 1 and 8 every 28 days, which was dose guided by blood counts per medical oncology. She also received two DEBIRI TACE procedures, and restaging imaging at three months showed considerable response to therapy, though with persistent right lobe disease and normal appearing left lateral segment (Figure 2). By mRECIST criteria, the patient had a large centrally necrotic tumor with nodular areas of enhancement consistent with viable tumor and partial response to liver-directed therapy. A right portal vein embolization was performed (Figure 3), and she was scheduled for exploration and resection approximately six weeks later. Post-embolization imaging demonstrated appropriate left lateral segment hypertrophy with expected liver remnant of 30%.

During the procedure, there was no evidence of carcinomatosis. Low central venous pressure management was employed. The lesion occupied the majority of the right liver lobe and extended through segment 4 of the left lobe adjacent to the falciform ligament. Intraoperative ultrasound was performed to ensure no disease in the expected liver remnant.

**Figure 4:**
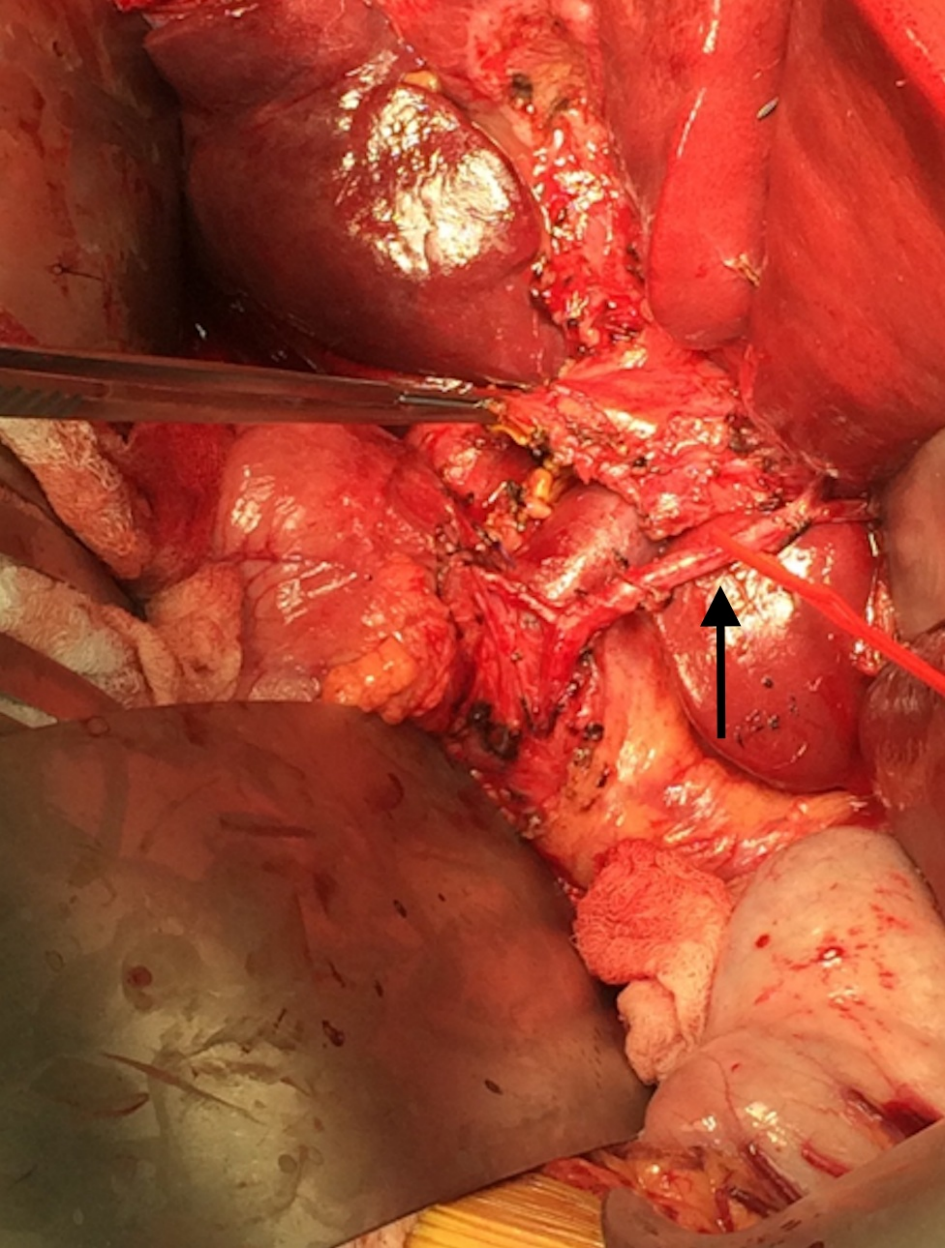
Portal dissection. Red vessel loop around segment 2/3 left hepatic artery. Portal vein visible, forceps retracting distal common bile duct.

**Figure 5:**
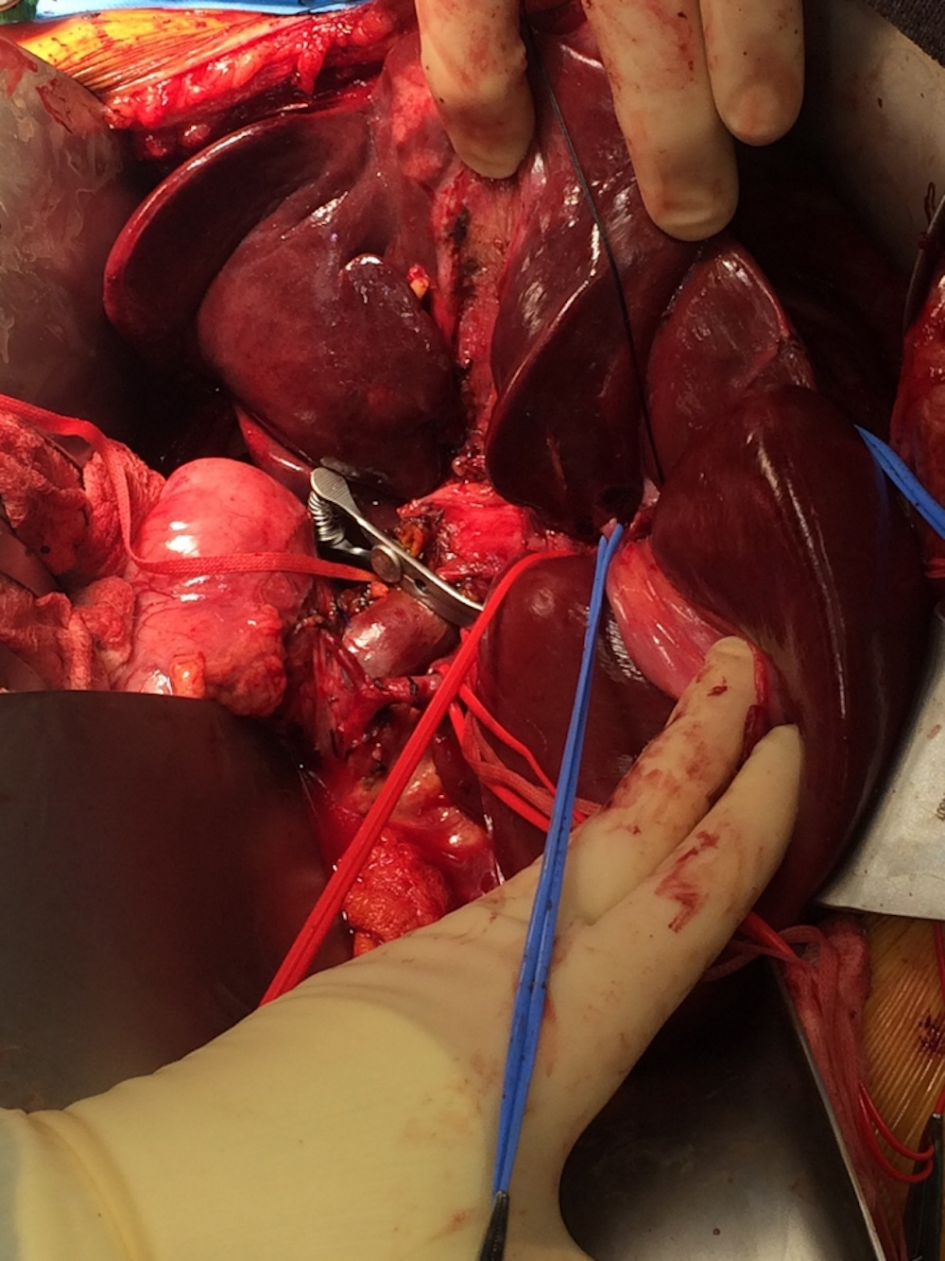
Falciform ligament and porto-umbilical fissure dissection. Ligation of segment 4 branches and preservation of segments 2 and 3 vascular pedicles.

The liver was completely mobilized from the retroperitoneal attachments. Complete portal and celiac lymphadenectomy was performed, the left hepatic artery to segment 2/3 was identified and preserved in addition to the distal branches of the left portal vein, which were carefully identified by dissection into the porto-umbilical fissure (Figures 4-5).

**Figure 6:**
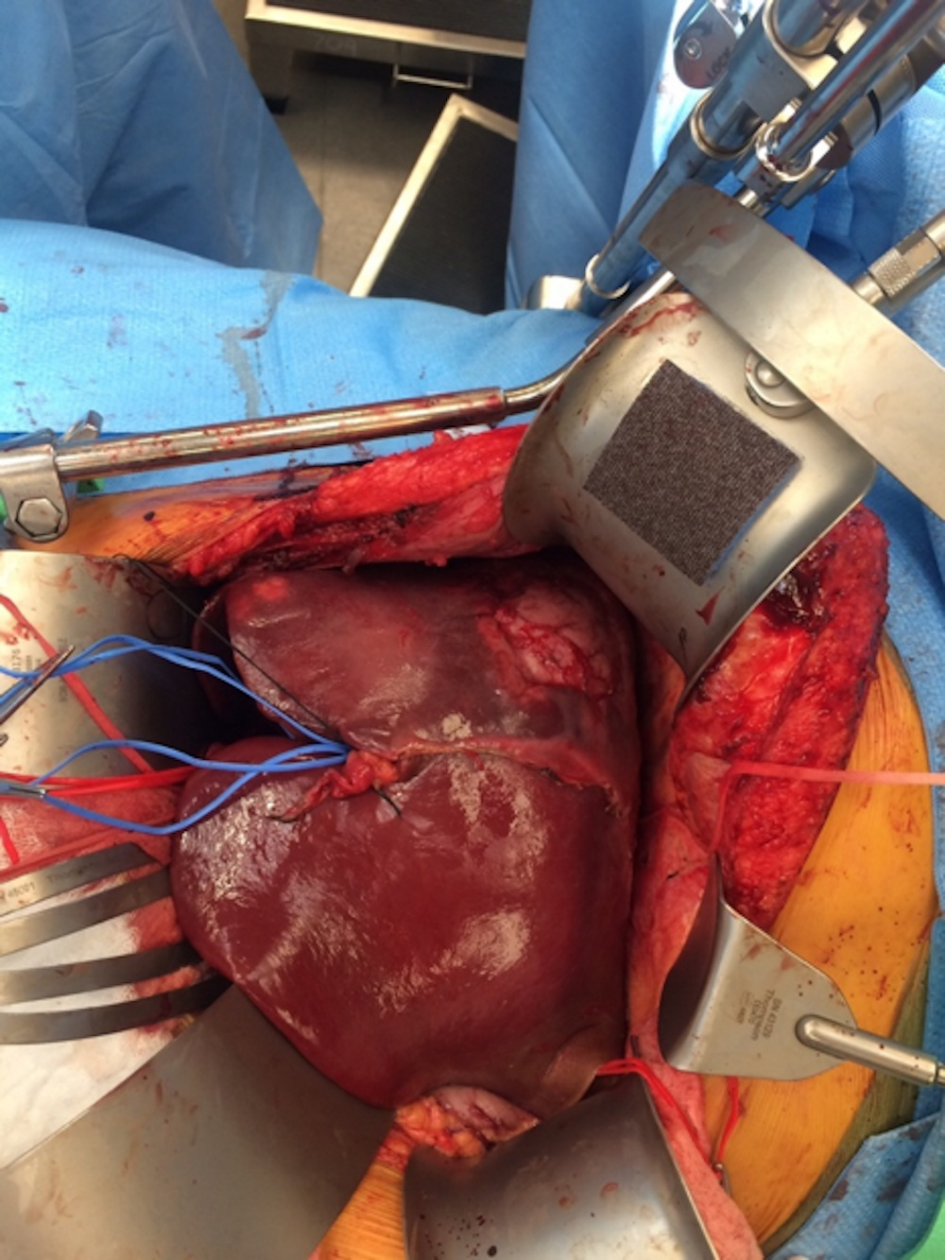
Demarcated segments 4-8 for extended right hepatectomy with extensive tumor burden in right lobe and medial sector of left lobe.

**Figure 7:**
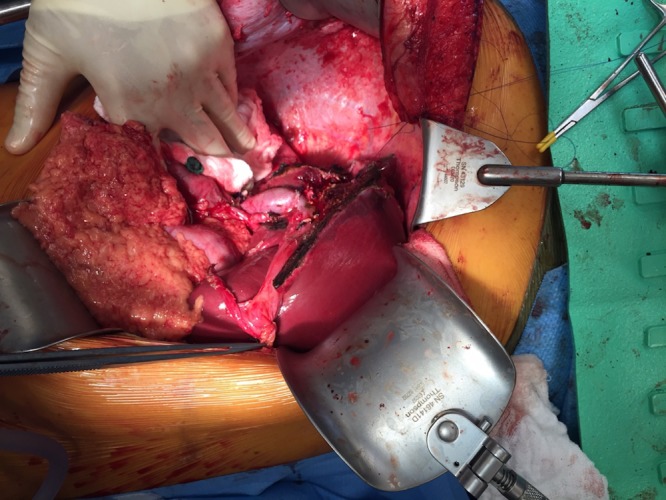
Extended right hepatic lobectomy completed with portal vein reconstruction visible.

**Figure 8:**
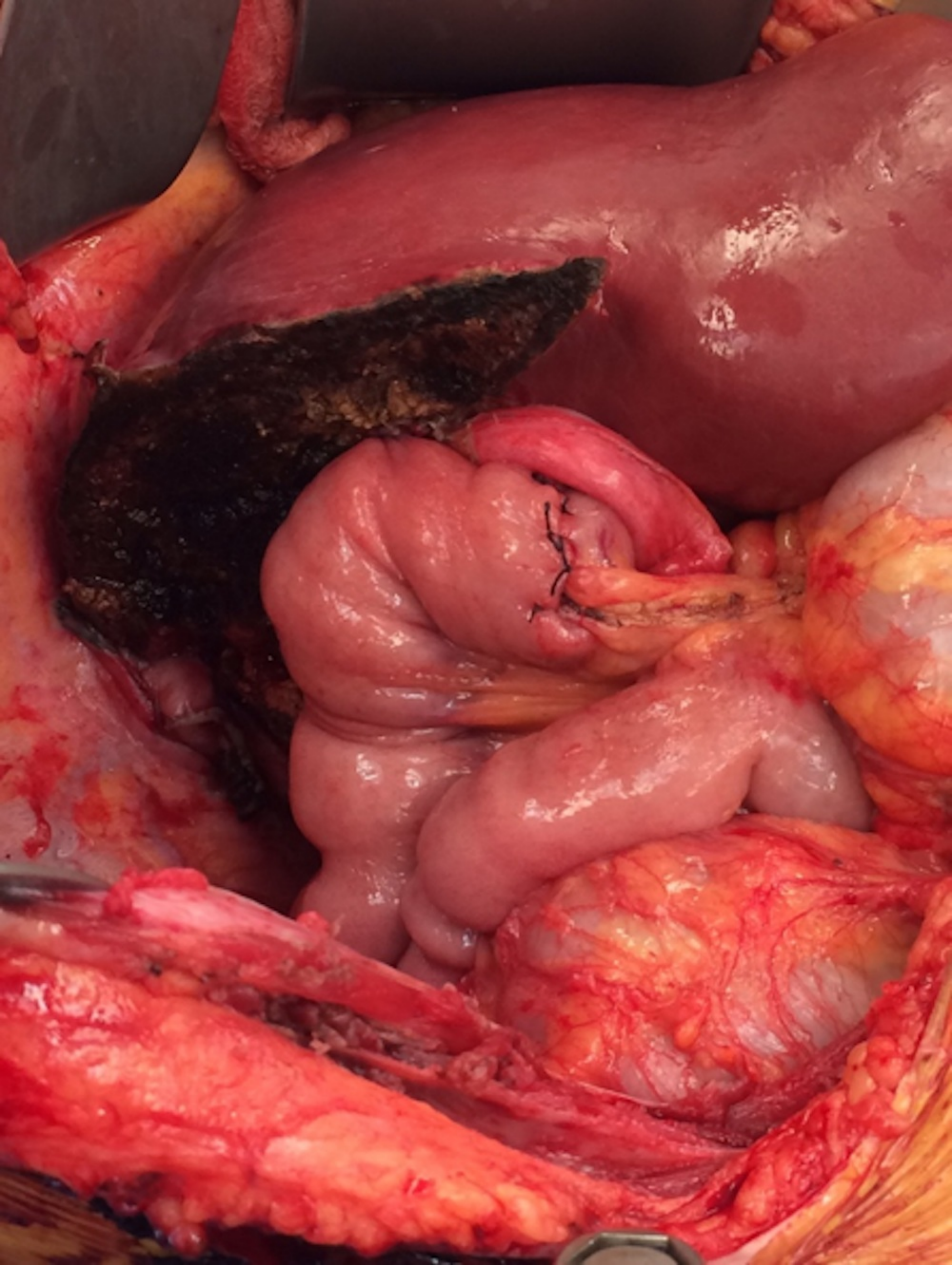
Completion of intrahepatic Roux-N-Y hepaticojejunostomy to segments 2 and 3 ducts.

Once resectability was confirmed, all segment 4 branches of the hepatic artery and portal vein were identified and ligated. The right hepatic artery and right portal vein were ligated. The distal common bile duct was transected, margin confirmed negative, and fluoroscopy confirmed appropriate location of the segment 2/3 ducts joining the left hepatic duct. The left, middle, and right hepatic veins were encircled. The liver was scored at the demarcation of segment 4 and segment 2/3 (Figure 6). The liver was transected using a Ligasure device, clips, and sutures for larger vascular radicles. Once the hilar plate was approached, it was clear that the tumor was abutting a portion of portal vein bifurcation. We elected to clamp the portal vein proximally and at the segment 2/3 bifurcation and resect the involved portion of the left portal vein, with primary reconstruction to the distal 2/3 portal vein branch. The specimen was completely transected and removed from the field after dividing the segment 2 and segment 3 bile ducts individually, the portal vein reconstruction was performed with running 6-0 prolene suture with a growth factor, and the liver remnant was reperfused (Figure 7). Two separate bile duct anastomoses were performed to a retrocolic Roux limb of jejunum for the segment 2 and segment 3 ducts using 6-0 PDS suture (Figure 8). Estimated blood loss for the entire procedure was 500 ml, no blood products were transfused. The patient was closed with one surgical Jackson-Pratt (JP) drain in the resection bed.

Postoperatively, the patient had an expected recovery with normalization of her liver enzymes. The patient has mild expected transaminitis following each of the two TACE treatments, which normalized within one week each. Peak lactic acid was 1.6 mmol/L (normal range 0.5-2.2 mmol/L), peak bilirubin was 2.2 mg/dL on post-op day 3, which returned to 0.8 mg/dL at the time of discharge. The remainder of her complete blood count (CBC) and electrolyte panels was within normal limits. With early ambulation, diet advancement, and pain control, the patient was discharged on post-op day 9.

**Figure 9:**
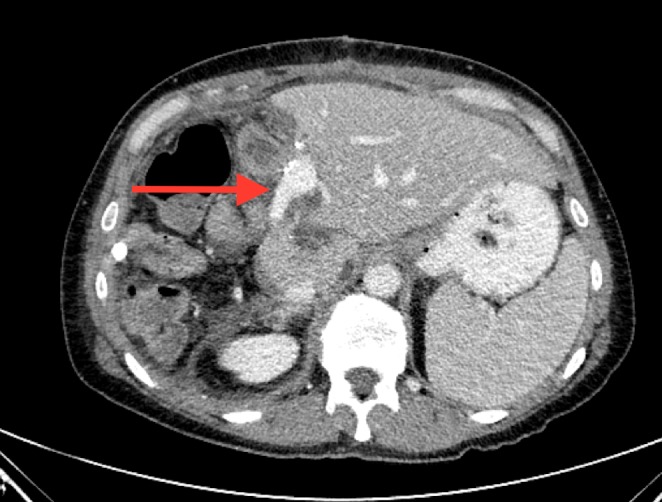
Axial image at post resection follow-up. Left lateral segment well perfused with appropriate hypertrophy. Left portal vein reconstruction visible.

At follow-up, the patient was doing well. Pathology revealed a moderately differentiated 10.5 cm cholangiocarcinoma with approximately 30% viable tumor with evidence of prior TACE treatment, negative surgical margins, 0/5 positive lymph nodes. Perineural and lymphovascular invasions were identified. The patient was followed for one year, with imaging at three-month intervals. The patient continued to be free of disease with interval surveillance imaging, shown at three months here (Figure 9). Due to the discussion at our multi-disciplinary tumor board regarding the negative surgical margins and node-negative disease, any further chemotherapy was withheld due to unclear benefit of further systemic therapy.

## Discussion

Historically, limited treatment options are available for iCCA and they have been associated with high rates of tumor recurrence and relatively short survival. The only potentially curative option is complete surgical resection, which has a reported five-year survival rate of 21-35%, though less than 30% of patients with iCCA undergo surgical resection [4]. Appropriate patient selection for attempted aggressive multi-disciplinary approach to management of “borderline-resectable” hepatic malignancies, particularly cholangiocarcinoma is key and includes performance status, optimizing nutrition, optimizing hepatic function preoperatively (biliary drainage if necessary for hyperbilirubinemia, percutaneous liver biopsy to r/o prohibitive liver disease or fibrosis, volumetric analysis of the expected liver remnant to avoid post-op liver failure), and ensuring no contraindications from a pulmonary or cardiovascular standpoint. Ideally, a young, otherwise healthy patient that can tolerate aggressive, multi-agent liver directed and/or systemic therapy as an adjunct to “downstaging” potentially unresectable tumors (i.e., shrinking, alleviating or preventing local or systemic spread). This patient was treated with TACE as well as systemic chemotherapy and portal vein embolization all of which encompassed approximately one year of therapy prior to pursuing surgical resection. This gives a “test of time” approach to the tumor biology to ensure no lymphadenopathy develops, no progression of disease, or any other factors that would preclude a safe resection. This is the same rationale that liver transplant for cholangiocarcinoma is used in very highly selected patients that receive chemotherapy, radiation therapy, and surgical node evaluation prior to proceeding with liver transplant, selecting out only the “good” patients that will benefit from liver transplant versus those with “bad tumor biology” that progresses and is non-transplantable, a very low percentage overall receives “curative” transplant.

Aside from surgical resection, liver transplant has been utilized as an attractive treatment option in well-selected patients, as the complete hepatectomy and biliary tree excision with lymphadenectomy confer the most durable chance of completely removing the tumor and tumor-bearing tissue. However, these patients must endure considerable morbidity associated with pre-transplant chemo and radiation therapy, and patients with decompensated liver disease concomitant with cholangiocarcinoma frequently are removed from transplant protocols due to inability to tolerate bridging therapy. Biliary drainage for obstructive jaundice in patients with unresectable CCAs and candidates for major hepatectomy with resectable CCAs has been advocated, though further studies would be beneficial in determining its exact role in all CCA patients as a consensus in management is currently lacking [2]. Various neoadjuvant therapies, such as systemic and regional chemotherapy, chemoembolization (TACE), radioembolization, stereotactic radiotherapy, clinical trials, and photodynamic therapy (PDT) have been utilized successfully in select patients, but the best evidence-based approach is still debatable. The chemotherapeutic regimen of gemcitabine plus cisplatin has been shown to be superior to other tested regimens. Leucovorin-modulated 5-Fluorouracil and capecitabine is a reasonable second-line alternative for patients who cannot tolerate the gemcitabine and cisplatin regimen.

Currently, ongoing clinical trials have examined the role of neoadjuvant therapies as a prelude to iCCA surgical resection, though no consensus has been reached as to the most effective approach. Neoadjuvant PDT has been shown to improve R0 resectability and survival when utilized in patients with primary unresectable pCCA prior to resection [5-7]. Without surgery, very few iCCA patients survive three years [3]. Any advancement in neoadjuvant therapy that would allow primary unresectable tumors to eventually be R0 resected would potentially improve the currently poor prognosis of iCCA.

## Conclusions

In conclusion, aggressive pre-operative neoadjuvant therapies, including systemic chemotherapy combined with liver-directed therapy, may be an effective strategy to provide curative surgical resection for well-selected advanced cholangiocarcinoma patients. However, more studies are warranted to determine the most effective treatment strategy for these rare but highly fatal tumors.
